# Utilizing Social Media to Study Information-Seeking and Ethical Issues in Gene Therapy

**DOI:** 10.2196/jmir.2313

**Published:** 2013-03-04

**Authors:** Julie M Robillard, Louise Whiteley, Thomas Wade Johnson, Jonathan Lim, Wyeth W Wasserman, Judy Illes

**Affiliations:** ^1^National Core for NeuroethicsDivision of Neurology, Department of MedicineUniversity of British ColumbiaVancouver, BCCanada; ^2^Medical Museion & Novo Nordisk Foundation Center for Basic Metabolic ResearchUniversity of CopenhagenCopenhagenDenmark; ^3^Centre for Molecular Medicine and TherapeuticsDepartment of Medical GeneticsUniversity of British ColumbiaVancouver, BCCanada

**Keywords:** gene therapy, social media, content analysis, ethics, public opinion

## Abstract

**Background:**

The field of gene therapy is rapidly evolving, and while hopes of treating disorders of the central nervous system and ethical concerns have been articulated within the academic community, little is known about views and opinions of different stakeholder groups.

**Objective:**

To address this gap, we utilized social media to investigate the kind of information public users are seeking about gene therapy and the hopes, concerns, and attitudes they express.

**Methods:**

We conducted a content analysis of questions containing the keywords “gene therapy” from the Q&A site “Yahoo! Answers” for the 5-year period between 2006 and 2010. From the pool of questions retrieved (N=903), we identified those containing at least one theme related to ethics, environment, economics, law, or society (n=173) and then characterized the content of relevant answers (n=399) through emergent coding.

**Results:**

The results show that users seek a wide range of information regarding gene therapy, with requests for scientific information and ethical issues at the forefront of enquiry. The question sample reveals high expectations for gene therapy that range from cures for genetic and nongenetic diseases to pre- and postnatal enhancement of physiological attributes. Ethics questions are commonly expressed as fears about the impact of gene therapy on self and society. The answer sample echoes these concerns but further suggests that the acceptability of gene therapy varies depending on the specific application.

**Conclusions:**

Overall, the findings highlight the powerful role of social media as a rich resource for research into attitudes toward biomedicine and as a platform for knowledge exchange and public engagement for topics relating to health and disease.

## Introduction

The field of gene transfer, often referred to as “gene therapy”, is rapidly evolving and generating hope for the treatment of a large variety of diseases and disorders [1,2]. Research developments and clinical trials are often featured prominently in traditional news media and on the Internet [3], and both media domains contribute to public expectations and health decision making. Existing alongside promises about the medical benefits of genetic research, which are often emphasized in news media [3], are ethical concerns about laboratory and clinical research and its translation into clinical settings [4,5]. Within the bioethics community, topics such as the risk-benefit tradeoff in human studies, potential inadvertent transmission of germline changes, the blurring of the distinction between research and treatment with attendant issues surrounding informed consent, and the possible use of gene therapy for nontherapeutic applications have been debated [4,5]. While these issues are covered prominently in the traditional news media and online when an event such as the death of a research participant occurs [6,7], little is known overall about how prospective patients and the broader public think about these concerns and indeed the extent to which they are concerned at all.

Our work draws on past studies that have polled public opinion regarding genetics research. In general, these survey studies have focused on testing existing basic scientific knowledge, perceived acceptability of treatments, and new scientific developments such as cloning. The results from these studies reveal ambivalence about gene therapy, and that acceptability is linked to the potential for treating serious diseases [8,9]. In a meta-analysis of survey research on various aspects of genetics, Singer showed that just over half of the respondents would be willing to undergo gene therapy, but that this measure of acceptability climbs to nearly 90% in the context of curing a fatal genetic disease in children or fetuses. Strong predictors of these attitudes are the degree of religious belief or practice [10]. These survey studies are all structured around fixed questions and thus do not capture emergent opinions or reflect participant-driven concerns. This is a particular concern for affective variables such as those related to ethics content, for which close-ended questions have low validity and risk creating framing effects, for instance by implying that an issue ought to be of ethical concern by asking if it is [11,12].

As people increasingly communicate through various forms of online media, it has become possible to use websites and online applications to assess freely initiated opinions and attitudes on a large variety of health-related topics [13,14]. Online social media hold particular potential for both the identification of attitudes and priorities when considering health interventions [15] and as a global, widely used, and accessible platform for engagement. As research investigating public attitudes and interactions through the lens of social media grows, various frameworks are emerging, such as narrative analysis of social media content [16]. Another such framework is infodemiology, the science of distribution and determinants of information in electronic media, including but not limited to the Internet and mobile applications [17,18]. The goal of infodemiology is ultimately to use the knowledge gained to inform public health and public policy. Examples of research using Internet parameters to survey health include the tracking of flu-related searches on an Internet search engine [19] and the examination of vaccine criticism on webpages [20]. The multidisciplinary field of infodemiology is emerging as a lens through which we can observe the health-seeking behaviors of people involved in social media and their attitudes towards health and illness [17].

Elucidating public perceptions of scientific research and clinical trials is crucial to a full understanding of contemporary biomedicine, which both influences and is influenced by public understandings of health, and which interacts with public opinion through mechanisms such as funding structures, patient advocacy, and protest, lobbying, and debate [21,22]. This broader understanding of the social context of research is also crucial for effective science communication. In recent decades, there has been much discussion of the failures of top-down dissemination of scientific knowledge to improve public trust in science, increase scientific literacy, or produce more engaged scientific citizens [23,24]. Effective communication relies on the diverse forms of knowledge, expertise, and attitudes that different public audiences bring to the conversation [23,24] and on taking seriously its multidirectional nature [25]. Though social and online media introduce concerns about accuracy, trust, and expertise [26-29], they offer new possibilities for communication and for breaking down traditional barriers between expert scientists and public audiences. Research into how social media are used is crucial to grounding future efforts to utilize these platforms to promote public engagement with biomedical research and its clinical application [30].

The proposed methodological shift from dissemination to dialogue has been motivated and accompanied by a normative argument about the political and ethical desirability of fully engaging public audiences in scientific research [23,25,31,32]. Arguments for reciprocal public engagement are particularly pressing for biomedical research, which can have a profound impact both on people’s health and on their sense of self and social relations and for clinical domains where decisions about treatment are rarely black and white. The present study delivers insights into current concerns and information-seeking practices surrounding gene therapy among users of a highly trafficked social media website. In doing so, the study adds to knowledge about the specific challenges of communicating genetics research [33,34] and contributes to a growing body of knowledge about the possibilities of social media for public engagement [27,29].

## Methods

### Study Design

We conducted a content analysis of questions containing the keywords “gene therapy” from the Q&A site *Yahoo! Answers* for the 5-year period between 2006 and 2010.

### Sample and Data Mining

We mined the online Q&A website Yahoo! Answers to obtain the sample of question and answers for this study. Yahoo! Answers is a website belonging in the social media family. It constitutes a social software that specifically supports interactive dialogue and user-generated content, blurring boundaries between media producer and consumer. Launched in 2005, Yahoo! Answers is a free, community-driven “knowledge market”. On the site, users can both submit questions to be answered and answer questions posed by other users, with a points system being used to encourage participation. While points have no value, they serve as an indicator of how active a user is, and reaching point thresholds (levels) can give a user more site access. For each question, a “Best Answer” is selected either by the asker or through votes by the community. Yahoo! Answers was chosen over other similar Q&A sites based on two criteria: (1) it was the Q&A site gathering the largest traffic (as measured by unique monthly visitors measured by analytics provider Compete) at the time of the study, and (2) returned the largest number of matches for a search of the keywords “gene therapy”. According to December 2010 estimates by Quantcast, an audience measurement provider, Yahoo! Answers traffic is made up of similar proportions of male (47%) and female (53%) users, with the most present age groups being 25-24 (23%), 35-44 (22%), and 18-24 (19%).

We used a customized automatic program retrieval method to search for “gene therapy” on the Yahoo! Answers result pages in the 5-year time period between January 1, 2006, and December 31, 2010. Data fields for questions, answers, and users from each page were parsed and stored. Duplicates and irrelevant retrievals were manually removed from the database.

### Coding and Intercoder Reproducibility

#### Coding the Questions

The first phase of the analysis considered the questions. The entire sample was coded by one investigator (JR), using a coding guide developed by 2 coders (JR and LW) from a pilot analysis of a random sample of 10% of the data. A second coder (TJ) analyzed 20% of the final sample to test for reproducibility, tested via percentage intercoder reliability. Reproducibility was initially 93%, and remaining disagreements were settled through discussion to achieve consensus.

We used an emergent coding strategy where categories were established after an initial, preliminary examination of 10% of the sample by two independent coders. The coding structure was developed to capture the salient thematic features of our sample as identified in our preliminary analysis. The final coding guide comprised the following major themes: (1) type of question (eg, request for scientific information, opinion gathering), (2) application of gene therapy (eg, disease treatment, enhancement), and (3) ethical, environmental, economic, legal, and social implications (eg, effects on self, impact on society) of gene therapy. We further coded for subthemes within each major theme. For an example of the coding strategy used for the question sample, see Table 1.

**Table 1:** Example of coding strategy (questions).

Theme	Subtheme^a^
Question features	
	Question ID
	Year
	User name
Theme 1 Type of question	
	Science information
	Effectiveness
	Progress
	Risks
	Ethical, environmental, economic, legal, social issues
	Education
	Careers
	Opinion
	Polemic
	Other (describe)
Theme 2 Applications of gene therapy	
	Disease treatment
	Disease cure
	Disease prevention
	Enhancement
	In utero
	Sexual orientation
	Other social modification (describe)
Theme 3 Ethical, environmental, economic, legal, social issues	
	Resource allocation
	Effects on self
	Change to society
	Discrimination
	Nature
	God and religion
	Evolution

^a^ Multiple subthemes within a theme may apply to a single question.

#### Coding the Answers

We analyzed answers given by users to the subset of questions that contained a theme related to ethics, environment, economics, law, or society (ie, those that received a code under Theme 3) of the coding guide for questions. This focus derived from our specific interest in the views of public users on ethical and societal implications of gene therapy, shaping the development of a second, answers-specific coding guide based on an initial analysis of 10% of the answers. Intercoder reproducibility was assessed in the same manner as for analysis of the questions. The thematic categories in the answer coding guide were (1) attitudes towards applications of gene therapy (eg, explicitly for or against enhancement), and (2) ethical, environmental, economic, legal, and social implications of gene therapy (eg, risk-benefit balance, diversity). We further coded for subthemes within the main themes using the same methods applied to the questions.

Questions and answers were the unit of analysis to which individual codes were applied. We used a rich coding strategy, allowing multiple categorizations of individual questions and answers [35]. The analyses of both questions and answers could be considered a form of mixed-methods research, utilizing an initial qualitative and theory-driven evaluation of a sample to produce a coding guide amenable to further quantitative content analysis. This approach is appropriate when the goal is to produce a representative picture of a large population but without wanting to determine the coding schema in advance [36].

### Statistical Analysis

We used descriptive statistics to quantitatively characterize the composition of the both the question and answer samples generated by the coding guide.

## Results

### Final Sample

**Figure 1:**
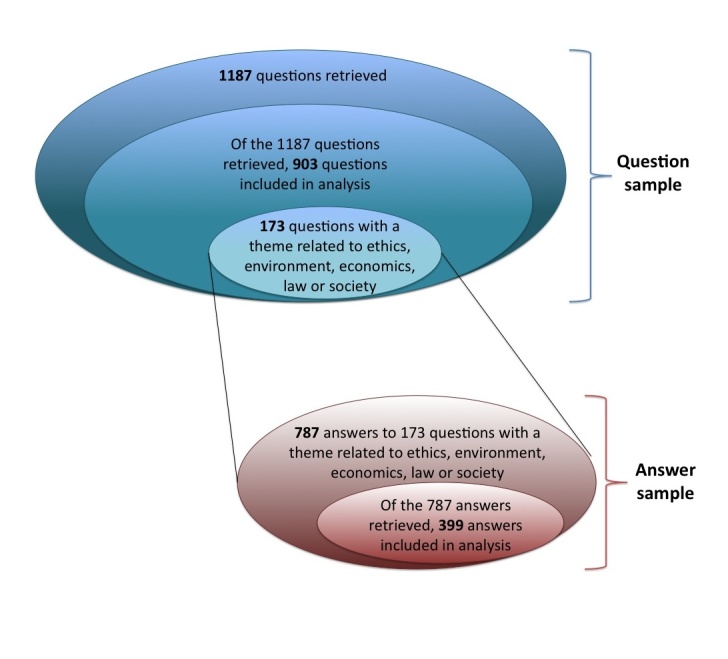
Data sample. Diagram showing the samples for questions and answers and the relationships between data sets.

The initial search for questions submitted to Yahoo! Answers between 2006 and 2010 and containing the keywords “gene therapy” yielded 1187 entries (see Figure 1 for a schematic of the sampling procedure). Duplicates and questions that did not discuss gene therapy were removed. The resulting final sample for the questions contained 903 entries. From this question sample, we retrieved 173 questions containing a theme related to ethics, environment, economics, law, or society for analysis. The initial answer sample from these 173 questions contained 787 entries. Following removal of answers that did not discuss gene therapy, the resulting answer sample contained 399 entries. We chose quotations that are representative of each analytic category to illustrate and discuss individual themes.

### Questions

#### Types of Questions

The sample of questions regarding gene therapy was diverse (see Table 2 for a breakdown and examples). Nearly half of the questions (49%) included a request for information about the science of gene therapy. These questions related to areas such as the methods for carrying out gene therapy and the process by which cells can express new genes. Other questions focused on possible ethical, environmental, economic, legal, and societal implications of gene therapy (19%), including references to god and religion, societal outcomes of gene therapy, and impact of gene therapy on the self. A similar proportion of questions (18%) was about the progress of gene therapy and probed whether gene therapy was currently available for different diseases. Questions directly probing the opinion of other users on various aspects of gene therapy made up 13% of the sample. Smaller subsets of the questions included requests for information about careers in gene therapy (3%), information about risks (3%), and information regarding the effectiveness of gene therapy, in which users often expressed knowledge about the availability of gene therapy but were unclear on efficacy (3%). A small proportion of questions (1%) was polemic in tone.

**Table 2:** Examples of types of questions.

Subtheme	Frequency (%)	Examples of questions	Year
Science	49	How do they [providers of gene therapy] change the genetic makeup in all the millions of cells?	2006
Education	44	Could ny1 giv me a short paragraph on 'Somatic cell gene therapy' for a gene therapy essay on cystic fibrosis	2007
GE^3^LS	19	Is it ok to build the perfect or elite human or is it only ok to fix genetic diseases such as alzimers?	2006
Progress	18	Is there any gene therapy available for parkinson’s disease?	2010
Opinion	13	If we have a gene therapy injectible, which would make you illness free, and comes free, would you go for it?	2007
Careers	3	hi, […] i really am interested in gene therapy. what would i have to take for […] post grad for a career in gene therapy?? i want to work in a lab and do research etc.... that kind of job. so what career options would be open for me?	2009
Risks	3	What are the risks involved in using gene therapy?	2006
Effectiveness	3	To what extend is gene therapy effective in treating cancerous diseases?	2008
Polemic	1	Modern Day Liberalism. Mental Illness or Mental Deficiency? I'm not sure witch one. I know the main syptom is intellectual laziness and the inability to see reality and thier surroundings. If its an illness than it should be able to be cured. If its a deficiancy than its genetic and cannot be cured without gene therapy. What do you think.	2006

#### Applications of Gene Therapy

**Figure 2:**
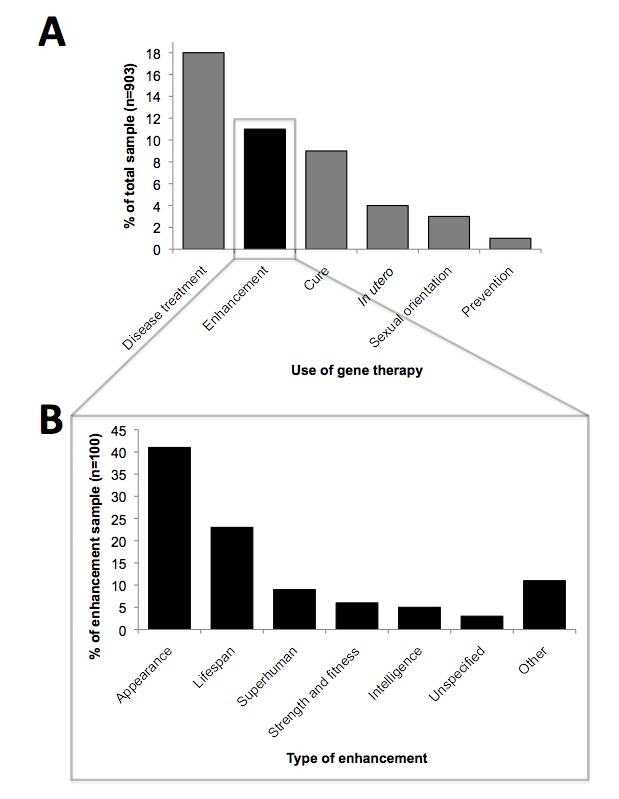
Uses of gene therapy. A) Codes for uses of gene therapy encountered in the question sample. B) Codes for the types of enhancement encountered in the question sample.

All questions were coded for mentions of specific applications of gene therapy (Figure 2). Applications focused largely on disease treatment or cure (27%). A fifth of all questions (20%) mentioned a specific disease or condition; 39 such diseases and conditions were recorded from the sample ranging from benign (acne) to severe (Alzheimer disease) (see full list in Multimedia Appendix 1). Of the questions mentioning a specific disease, 25% were about cystic fibrosis, 15% were about various forms of cancer, and 7% were about diabetes, with other diseases represented by smaller portions.

Other questions mentioned nontherapeutic applications of gene therapy, such as enhancement performed in children or adults or *in utero*. Figure 2 illustrates the subthemes within enhancement, with the most common being enhancement of physical appearance. Other forms of enhancement included increasing lifespan and unusual types of enhancement, such as gaining superhuman characteristics (eg, wings, chloroplasts) (Table 3).

**Table 3:** Examples of types of enhancement.

Theme	Example	Year
Appearance	Would you have gene therapy if it really could make you look 18 again?	2007
Lifespan	A genetic researcher stated that […] gene therapy will drastically increase human life spans. Assume he is correct, how would life change?	2007
Superhuman	With today’s advances in gene therapy, can people ever expect to have wings?	2006
Strength and fitness	Is there a gene therapy that can make you run faster?	2010
Intelligence	In the future, will it be possible to make humans more intelligent...? With gene therapy? I’ve heard about this, they’re already thinking about trying it with mentally retarded children, and in the future they may well be able to do it with adults as well.	2007
Unspecified	Now that we are in the age of gene therapy and genetic engineering, would you gene boost yourself?	2007

#### Ethical, Environmental, Economic, Legal, and Social Implications

We further coded all questions referring to ethical, environmental, economic, legal, and societal implications of gene therapy (19% of total sample, see above section on Types of Questions). The major subthemes (Table 4) were the impact of gene therapy on society (5% of total sample), on the self (4%), and god and religion (4%). Other subthemes were resource allocation (2%), evolution (1%), nature (1%), and discrimination/equity (1%).

**Table 4:** Examples of ethics, environment, economic, and legal themes in questions.

Theme	Example	Year
Change to society	[…] With gene therapy and other genetic research going on out there I’m sure scientists will know how to turn off the “aging gene or genes” sooner or later. […] I’m sure there are benefits and unforseen side effects on the human body and in civilization in general.	2007
Effects on self	Do you think the tendency toward fundamentalism is genetic? If so, could gene therapy wipe this disease out […]?	2007
God and religion	Do atheists need genetic therapy? There has been a discovery of a “faith” gene in scientific research that theorizes faith could be genetic. Could this be the “mark” that is talked about in the Bible?	2010
Resource allocation	[…] I have to have 3 bioethical issues to discuss, and I can’t find anything other than, death from trials of gene therapy, and that only the rich can benefit. […]	2009
Discrimination and equity	[…] But if scientists were to take [gene therapy] a step further and maybe alter genes to have perfect vision, better immune system, change genes to make a child taller, etc. If this was done successfully and it was applied to many child or embryos, would it be right to say that child that did not have gene therapy to get superior genes be inferior in a way? Then could they be considered to be a sub-class of humans […]?	2008
Against nature	If you were pregnant and found out your baby was going to be retarded, […] and the doctor told you that with experimental gene therapy it could be fixed while still in the womb by manipulating a single gene, would you do it? Or would you let nature run its course?	2009
Evolution	How cloning, gene therapy, and other technologies affect evolution?	2009

### Answers

In the second phase of analysis, we studied the answers given to the subset of questions referring to ethical, environmental, economic, legal, and societal implications of gene therapy (n=173; 19% of total sample). Below we first characterize the number of answers generated by these questions and then report the coding results under the major themes of the analysis: (1) attitudes towards applications of gene therapy, and (2) ethical, environmental, economic, legal, and social implications of gene therapy.

#### Answer Statistics and Response-Generating Topics

Questions containing a theme related to ethical, environmental, economic, legal, or societal implications of gene therapy (n=173) had a mean of 4.5 answers each (range: 0 to 25 answers/questions). Nearly two thirds of the questions (63%) had 1-3 answers. We retrieved the questions that generated the top 1% of number of answers/question (≥ 17 answers/question). Ten questions from our sample (10/173) met this criteria. Of these 10 questions, 7 were about gene therapy to modify sexual orientation; the other 3 were about gene therapy for longevity, gene therapy for enhancement in adults, and gene therapy for enhancement *in utero*.

#### Attitudes Towards Applications of Gene Therapy

**Figure 3:**
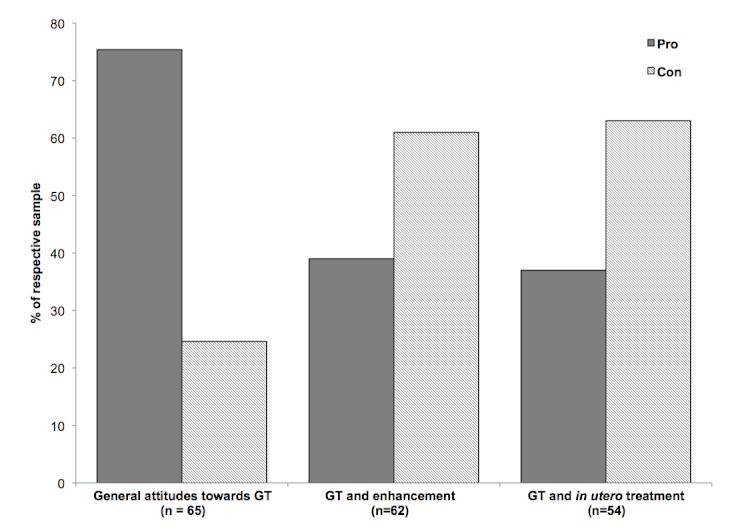
Attitudes towards gene therapy. General attitudes towards applications of gene therapy encountered in the answer sample.

Users expressed general attitudes about gene therapy in 65 of the answers (Table 4), of which 75% answers were in favor of (for) gene therapy. Out of the 38 answers in which attitudes were instead expressed specifically regarding nontherapeutic enhancement (eg, a use of gene therapy not aimed at treating or curing diseases, but rather at enhancing human characteristics such as appearance or intelligence), only 39% of users were in favor. Various arguments were stated both for and against gene therapy, which we identified through the positive or negative valence of the answers. Examples of these arguments (Table 5) included making the most of emerging technologies and abiding by religious rules. The proportions for acceptability were similar in the case of enhancement *in utero* (Figure 3).

In addition to these attitudes about applications of gene therapy, answers revealed attitudes regarding ethical aspects of gene therapy. In this context, a majority of answers (>50%) suggested that gene therapy was against nature, that it was not against religion, that it held the potential to control evolution, and that it would lead to discrimination and inequity as well as to uneven resource allocation.

**Table 5:** Examples of attitudes towards gene therapy and its applications.

Theme	Example	Year
For gene therapy	Support it [gene therapy], it could save lots of lives and maybe even yours one day.	2009
Against gene therapy	[…] The last thing we need to do is to genetically alter natural life.	2009
For enhancement	Cobble a gene together and create a new characteristic for man. That is the future. I hope I will be able to get some cool characteristic like a sonar.	2006
Against enhancement	We wish to go back to heaven where we once belong, so preserving/cure human health is allowed, but enhancing it may mostly seen unethical.	2007

#### Ethical, Environmental, Economic, Legal, and Social Implications of Gene Therapy

While some ethical issues that arose in the question sample were also present in the answer sample (eg, impact on self and on society), new ethical issues emerged in the answers. We describe here a few qualitative examples from these themes that appeared in only a few instances. While in the questions users asked about risks of gene therapy, in the answers users responded by demonstrating reasoning about the dependence of the risk-benefit calculation on the availability of other options (1%): “[…] Although, cancer is generally treatable whereas X-SCID is fatal so it’s not necessarily the end of the world if someone gets cancer since there are options” (2008). Other new concepts included conformity and diversity (2%), as some users saw the advent of gene therapy to be a threat to genetic diversity: “Imagine if everyone could create the kid of their dreams. There would be nothing on this planet but perfect-looking human beings. And imperfect on the inside…” (2007). As well, the answers sample discussed issues around freedom of choice (2%), as some users felt gene therapy might become mandatory for certain conditions: “[…] Fixing birth defects is cool, but government interference in our baby kids is just less freedom” (2009).

## Discussion

### Principal Results

This content analysis of questions and answers about gene therapy from a major online social media platform provides new insights into public discourse on gene therapy. The results show that (1) social media users are seeking a large variety of types of information regarding gene therapy and, after requests for scientific information, the cluster of ethical, economic, environmental legal, and social issues are at the forefront of the discussion, (2) questions about gene therapy reflect high expectations that range from cures for a large number of diseases—both genetic and nongenetic—to enhancing various physiological features before and after birth, (3) fears primarily concern changes to self and society, and (4) the acceptability of gene therapy varies depending on specific applications.

### Comparison With Prior Work

These results are consistent with those of an international survey about gene therapy in which 75% of responders supported the personal use of gene therapy, but significantly fewer supported specific applications involving nontherapeutic enhancement [37]. Those data and others suggest that acceptability of human genetic manipulation is weighed according to the perceived benefits and risks of the intervention [9]. Another previous study, examining prospective attitudes to gene therapy among patients uncovered significant concerns about effects on personal identity [38], again echoing the findings of the present study. Studies looking at genetic testing and engineering report that religious practice and beliefs are predictors of attitudes towards these technologies [10], consistent with the prominent religion theme in the present sample.

Better information on public attitudes is crucial to informing debate about the meaning, directions, and applications of biomedical research, augmenting academic discussion with public and stakeholder voices [24,31,34,39,40]. This study joins a growing body of research utilizing the potential of social media to capture such voices without the constraints of close-ended questionnaire research, or the problems of access and resources that are attendant to in-person ethnographic or sociological research [14,15]. Research harnessing the multidirectional features of online communications may take various forms, such as the tracking of the distribution and determinants of information online (infodemiology) [17-19], narrative analyses of social media content [16], computer-assisted data crawling [41], and the assessment of the impact of using social media platforms such as blogs on experiences of illness [42]. These emerging frameworks and methods are aimed at both better understanding, and ultimately improving, health and health policy, and they constitute a reflection of the growing role of social media in health communication [30].

By studying user-generated content, we were able to demonstrate high levels of interest in gene therapy—and in its potential environmental, economic, legal, and social implications*—*without intervening with a clipboard or audio recorder. Although the disadvantage of this hands-off approach is that we were not able to probe users’ perspectives directly, we were able to sample a diverse range of apparent motivations for asking and answering questions. These included a demonstrated interest in particular diseases, dissatisfaction with physical appearance, concerns about procreation, educational needs, and, interestingly, a desire to generate debate outside of institutionalized, top-down science communication frameworks. We uncovered concerns around nontherapeutic enhancement, including discussion about impact on selfhood and authenticity—a topic deserving further research. The spontaneous nature of communication on social media thus gives insight into public interests and attitudes, and the diversity of information-seeking practices. Its participatory format also suggests possibilities for enriching public engagement. For instance, there was a high level of demand for scientific information. This finding supports arguments made in the context of Wikipedia and blogging for scientific voices to join in online discussion and information curation, rather than just posting static texts on institutional websites [29]. Overall, this study emphasizes the value of taking social media seriously—even Q&A websites, which are often criticized for disseminating inaccurate information. In particular, the prominence of spontaneous debate of ethical issues suggests that such platforms are a good setting for public engagement, augmenting more formal, top-down practices such as the consensus conference or public panel debate [43].

Despite its promise, online public engagement with health information and biomedical research raises concerns. Scientists keen to garner public trust and support, health care providers worried about poor decision making, or educators trying to promote evidence-based learning, all bemoan the “wild west” qualities of the Internet [44]. An increasing amount of research has highlighted shortcomings of online health information, ranging from uneven quality of medical information to the potential for harm and the risks of overconsumption of health information [44]. More work needs to be done to assess the current state of online information and how it can be made more accurate, relevant, and open. This study argues for attending to what people seek to know in this endeavor and to the active nature of intra-user information sharing over traditional source-to-receiver model of information transmission. Our observation that users often reasoned according to the social context and possible alternatives of particular applications, rather than giving a universal risk-benefit analysis, emphasizes the importance of elucidating context in public engagement activities.

Analysis of traditional media forms has often focused on the way in which research is framed and the potential effects on public opinion and health decision making [45]. For instance, news reports frequently frame stories about genetics in a way that emphasizes the potential benefits of genetic research [3]; as another example, deep brain stimulation is often reported in the form of miracle stories that lack discussion of ethical issues [46]. As Johansson (2011) points out, however, optimism and fears may be represented in different media information channels, resulting in an array of perspectives that may not be fully integrated [47]. When discussing the impact of a particular media form, it is thus important to remember that it is only one among many. For instance, we speculate that important media contexts for gene therapy include the positive framings of future treatments in the news media, dystopian fictional representations of cloning, the wide circulation of religious concepts in secular contexts, and a broader culture that increasingly views the physical body as a malleable platform for shaping the self [22]. Q&A websites themselves seem to be characterized by a diverse range of framings of gene therapy and situate ethics discussion within more pragmatic information-seeking and in relation to a range of positions and styles of reasoning.

### Limitations

Despite the attractions of using social media as a research tool, we also appreciate the limitations of this study. Research using social media lends itself to selection bias, as it can be difficult to establish whether the study population represents the sampling population [48]. Our answer sample is also susceptible to response bias, especially when the question contains an explicit opinion that may direct and frame the discussion. While these biases inevitably limit the generalizability of our results and call for replication and convergent evidence, this study provides specific insight into the information-seeking patterns and the attitudes of the large Yahoo! Answers community.

Another limitation relates to our sample: it derives from a single social media platform, and while Yahoo! Answers boasts high traffic and relatively broad demographics, it may not represent public attitudes as a whole, including those of nonInternet users. Nonetheless, the consistency of the present findings with related studies on attitudes to gene therapy suggests that Yahoo! Answers attracts a sufficiently broad segment of the population to act as a proxy for public opinion at this level of analysis. We recognize that in aiming for a generalizable sample of public attitudes, we are not attending to the more specific interests and expertise that particular user-groups such as patients, religious groups, or health professionals might bring to this topic. The absence of reliable demographic information on Yahoo! Answers also poses a limitation. While it protects the anonymity of users, it makes it impossible to verify the authenticity of content. As well, Yahoo! Answers users are not required to volunteer sociodemographic details such as age or gender, and our study design does not involve contacting the users in any way, thus limiting our ability to undertake statistical analyses by age, gender, or other demographic characteristics to establish correlates of attitudinal findings. Finally, we cannot confirm that Yahoo! Answers users possess a clear understanding of what constitutes gene therapy. However, as our interest is in interests and opinions rather than in the accuracy of public knowledge, it is appropriate that we examine what users understand as gene therapy.

### Conclusions

Overall, we find a rich discussion of gene therapy and associated ethical issues on a social media platform, which represents a spontaneous form of public engagement but also highlights a need for improved communication about gene therapy. The present work and future studies in this area are critical to inform research and medical communities of the current state of information-seeking and discussion regarding fast-paced advances in their fields and highlight the need for evidence-based and reciprocal communication between the academy and diverse publics.

## Multimedia Appendix 1

Diseases and conditions mentioned in question sample.

## References

[1] Bainbridge JW, Smith AJ, Barker SS, Robbie S, Henderson R, Balaggan K, Viswanathan A, Holder GE, Stockman A, Tyler N, Petersen-Jones S, Bhattacharya SS, Thrasher AJ, Fitzke FW, Carter BJ, Rubin GS, Moore AT, Ali RR (2008). Effect of gene therapy on visual function in Leber's congenital amaurosis. N Engl J Med.

[2] Wilson, JM (2011). The History and Promise of Gene Therapy. Genetic Engineering & Biotechnology News.

[3] Petersen A (2001). Biofantasies: genetics and medicine in the print news media. Soc Sci Med.

[4] King NM, Cohen-Haguenauer O (2008). En route to ethical recommendations for gene transfer clinical trials. Mol Ther.

[5] Rabino I (2003). Gene therapy: ethical issues. Theor Med Bioeth.

[6] Couzin J, Kaiser J (2005). Gene therapy. As Gelsinger case ends, gene therapy suffers another blow. Science.

[7] Savulescu J (2001). Harm, ethics committees and the gene therapy death. J Med Ethics.

[8] Calnan M, Montaner D, Horne R (2005). How acceptable are innovative health-care technologies? A survey of public beliefs and attitudes in England and Wales. Soc Sci Med.

[9] Macer DR (1992). Public acceptance of human gene therapy and perceptions of human genetic manipulation. Hum Gene Ther.

[10] Singer E, Corning A (1998). The polls--trends: Genetic testing, engineering, and therapy. Public Opinion Quarterly.

[11] Barr M, Rose D (2008). The great ambivalence: factors likely to affect service user and public acceptability of the pharmacogenomics of antidepressant medication. Sociol Health Illn.

[12] Shaughnessy J (2005). Research Methods in Psychology, 7th ed.

[13] O'Connor B, Balasubramanyan R, Routledge BR, Smith NA (2010). From tweets to polls: Linking text sentiment to public opinion time series. Proceedings of the International AAAI Conference on Weblogs and Social Media.

[14] Scanfeld D, Scanfeld V, Larson EL (2010). Dissemination of health information through social networks: twitter and antibiotics. Am J Infect Control.

[15] Kozinets RV (2009). Netnography: Doing Ethnographic Research Online.

[16] Chou WY, Hunt Y, Folkers A, Augustson E (2011). Cancer survivorship in the age of YouTube and social media: a narrative analysis. J Med Internet Res.

[17] Eysenbach G (2009). Infodemiology and infoveillance: framework for an emerging set of public health informatics methods to analyze search, communication and publication behavior on the Internet. J Med Internet Res.

[18] Eysenbach G (2011). Infodemiology and infoveillance tracking online health information and cyberbehavior for public health. Am J Prev Med.

[19] Eysenbach G (2006). Infodemiology: tracking flu-related searches on the web for syndromic surveillance. AMIA Annu Symp Proc.

[20] Zimmerman RK, Wolfe RM, Fox DE, Fox JR, Nowalk MP, Troy JA, Sharp LK (2005). Vaccine criticism on the World Wide Web. J Med Internet Res.

[21] Bucchi M (2004). Science in society: an introduction to social studies of science.

[22] Rose N (2007). The politics of life itself: biomedicine, power, and subjectivity in the twenty-first century.

[23] Wilsdon J, Willis R (2004). See-Through Science.

[24] Gregory J, Miller S (2000). Science in public: communication, culture, and credibility.

[25] Racine E, Bar-Ilan O, Illes J (2005). fMRI in the public eye. Nat Rev Neurosci.

[26] Bucchi M (2008). Handbook of public communication of science and technology.

[27] Mandavilli A (2011). Peer review: Trial by Twitter. Nature.

[28] Rodder S, Franzen M, Weingart P (2011). The Sciences' Media Connection. Public Communication and Its Repercussions.

[29] Wolinsky H (2011). More than a blog. EMBO reports.

[30] Chou WS, Hunt YM, Beckjord EB, Moser RP, Hesse BW (2009). Social media use in the United States: implications for health communication. J Med Internet Res.

[31] Pickersgill MD (2011). Research, engagement and public bioethics: promoting socially robust science. J Med Ethics.

[32] Taylor PL (2007). Rules of engagement. Nature.

[33] Einsiedel EF, Geransar R (2009). Framing genetic risk: trust and credibility markers in online direct-to-consumer advertising for genetic testing. New Genetics and Society.

[34] Einsiedel EF, Jones M, Brierley M (2011). Cultures, Contexts and Commitments in the Governance of Controversial Technologies: US. UK and Canadian Publics and Xenotransplantation Policy Development. Science and Public Policy.

[35] Denzin NK, Lincoln YS (2005). The SAGE Handbook of Qualitative Research.

[36] Tashakkori A, Creswell JW (2007). Editorial: The New Era of Mixed Methods. Journal of Mixed Methods Research.

[37] Macer DR, Akiyama S, Alora AT, Asada Y, Azariah J, Azariah H, Boost MV, Chatwachirawong P, Kato Y, Kaushik V (1995). International perceptions and approval of gene therapy. Hum Gene Ther.

[38] Scully JL, Rippberger C, Rehmann-Sutter C (2004). Non-professionals' evaluations of gene therapy ethics. Soc Sci Med.

[39] Bubela T, Hagen G, Einsiedel E (2012). Synthetic biology confronts publics and policy makers: challenges for communication, regulation and commercialization. Trends Biotechnol.

[40] Greenberg AJ, McCormick J, Tapia CJ, Windebank AJ (2011). Translating gene transfer: a stalled effort. Clin Transl Sci.

[41] Doing-Harris KM, Zeng-Treitler Q (2011). Computer-Assisted Update of a Consumer Health Vocabulary Through Mining of Social Network Data. J Med Internet Res.

[42] Ressler PK, Bradshaw YS, Gualtieri L, Chui KK (2012). Communicating the experience of chronic pain and illness through blogging. J Med Internet Res.

[43] Horst M, Cheng D (2008). In Search of Dialogue: Staging Science Communication in Consensus Conferences. Communicating Science in Social Contexts: New models, new practices.

[44] Benigeri M, Pluye P (2003). Shortcomings of health information on the Internet. Health Promot Int.

[45] Nisbet MC (2004). Public Opinion About Stem Cell Research and Human Cloning. Public Opin Q.

[46] Racine E, Waldman S, Palmour N, Risse D, Illes J (2007). "Currents of hope": neurostimulation techniques in U.S. and U.K. print media. Camb Q Healthc Ethics.

[47] Johansson V, Garwicz M, Kanje M, Röcklinsberg H, Schouenborg J, Tingström A (2011). Beyond Blind Optimism and Unfounded Fears: Deep Brain Stimulation for Treatment Resistant Depression. Neuroethics.

[48] Janssens ACJW, Kraft P (2012). Research conducted using data obtained through online communities: ethical implications of methodological limitations. PLoS Med.

