# Supplementary material for: Utilizing Social Media to Study Information-Seeking and Ethical Issues in Gene Therapy
Source: J Med Internet Res. 2013 Mar 4;15(3):e44. doi: 10.2196/jmir.2313 (PMC3636301; doi:10.2196/jmir.2313)
Supplement: Supplementary file 1 [file jmir_v15i3e44_app1.pdf]

Appendix 1. Diseases and conditions mentioned in question sample.

Disease or condition	
Acne	Kidney failure
Adrenoleukodystrophy	Klinefelter syndrome
Albinism	Lactose intolerance
Alcoholism	Leber congenital amaurosis
Alzheimer disease	Limb-girdle muscular dystrophy
Cancer	Lymphedema
Celiac disease	Maple syrup urine disease
Cleft lip	Mental retardation
Cold sores	Myalgic encephalomyelitis
Color blindness	Obsessive-compulsive disorder
Common cold	Paget disease
Cystic fibrosis	Parkinson disease
Degenerative disc disease	Porphyria
Diabetes	Psoriasis
Dilated cardiomyopathy	Retinitis pigmentosa
Down syndrome	Rett syndrome
Dwarfism	Rh disease
Dyslexia	Scars
Erectile dysfunction	Schizophrenia
Fatal familial insomnia	SCID
G6PD deficiency	Sickle cell anemia
Hemophilia	Tongue thrust disorder
HIV	Turner syndrome
Hurler syndrome	
